# Accidental Ingestion of Tetrahydrocannabinol-Laced Gummies Causing Bradycardia and First-Degree Atrioventricular Block in a Pediatric Patient: A Case Report

**DOI:** 10.7759/cureus.26826

**Published:** 2022-07-13

**Authors:** Isra Idris, John R Diez, Betty Ansong Assoku, Susan Beker

**Affiliations:** 1 Pediatrics, Woodhull Medical Center, New York City, USA; 2 Pediatric Cardiology, New York University, New York City, USA

**Keywords:** bradyarrhythmia, marijuana, unintentional cannabis ingestion, first-degree av block, bradycardia

## Abstract

An increasing trend of cannabis use places children at risk for the detrimental effects of marijuana. Poison control centers in the United States have been experiencing an upsurge in calls involving marijuana ingestion among children in the past years, specifically in states where marijuana is legal. With marijuana ingestion, neurologic symptoms predominate but cardiovascular manifestations have also been observed. Bradycardia and bradyarrhythmia are both uncommon cardiac manifestations of cannabis ingestion in children. Here, we present the case of a previously healthy two-year-old male with sinus bradycardia and first-degree atrioventricular (AV) block following accidental ingestion of tetrahydrocannabinol-laced gummies. Although bradycardia and first-degree AV block are uncommon after cannabis ingestion in children, clinicians should be aware of these findings and must consider evaluating for marijuana toxicity whenever presented with these acute signs. Prevention is crucial and can be achieved through supervision, parental education, and support.

## Introduction

According to the World Drug Report 2021, around 4% of the global population uses cannabis (marijuana), making it the most widely used illicit drug worldwide [1]. With increasing cannabis use and the availability of different products including “edibles,” exposure of children to this harmful substance has become a public health concern [2]. Poison control centers in the United States have been receiving an increased number of calls involving marijuana ingestion among children in the past few years, specifically in states where marijuana is legal [3]. Symptoms range from somnolence or agitation to severe manifestations such as respiratory depression and coma [4-8].

While the psychoactive effects of cannabis are well known, the cardiovascular effects are not fully understood, especially in children. Heart rate, rhythm, and blood pressure abnormalities are some of the reported signs of toxicity [4,5,9], which were thought to be brought about by the activation of cannabinoid receptors in the cardiovascular system and autonomic nervous system [10-12]. Tachycardia is the most common cardiac manifestation of cannabis exposure but there are very few cases of bradycardia and arrhythmia reported in the literature [4,5,9]. Here, we present the case of a two-year-old child who had bradycardia associated with a first-degree atrioventricular (AV) block following cannabis ingestion.

## Case presentation

A previously healthy two-year-old male was brought by his mother to the emergency department for drowsiness. One hour prior to the presentation, his mother witnessed the patient eating her cannabis gummies. The number of cannabis gummies ingested was unknown. After an hour, the patient was noted to be increasingly sleepy and brought to the hospital. He had no fever, no dyspnea, no vomiting or diarrhea, no changes in urine output, and no seizures or abnormal movements. The mother denied any trauma.

The child was born term without complications. He had normal growth and development and was up-to-date on his immunizations. There were no prior hospitalizations, surgeries, or recent travel. The patient did not have any diseases and was not on any medication. The mother reported no history of hematologic, oncologic, neurologic, metabolic, or renal disease in the family. There was also no family history of arrhythmia or sudden death.

The patient lived with his mother, a four-year-old sister, and nine other relatives in an apartment. His mother and two other adults smoked cigarettes and marijuana inside the house but stated that they always placed the children in a separate room when they smoked. The mother denied any present or past history of domestic violence at home and denied the use of illicit substances among household members.

Upon arrival at the emergency department, the patient was weak and drowsy. He was afebrile, normotensive (95/65 mmHg), bradycardic for age (heart rate range: 65-78 beats per minute), and with a normal respiratory rate (37 cycles per minute). The glucose level was normal (103 mg/dL). On physical examination, the patient had no evidence of trauma and no signs of dehydration. Pupils were 2 mm and equally reactive. An examination of the lungs and abdomen was normal. On cardiac examination, the rhythm was regular, with no extra heart sounds, no murmurs, and the point of maximal impulse was at the fourth intercostal space left midclavicular line. Pulses were equal, and capillary refill was less than two seconds. There was no cyanosis or any skin lesions.

**Figure 1:**
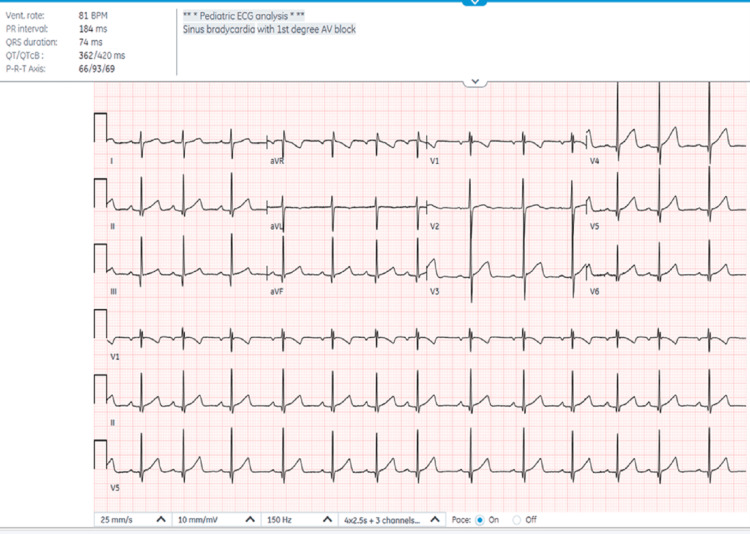
A 12-lead EKG showing sinus bradycardia with first-degree AV block (prolonged PR interval of 184 ms with 1:1 AV conduction). EKG: electrocardiogram; AV: atrioventricular

Complete blood count, electrolytes, blood glucose, creatinine, liver enzymes, bicarbonate, ethanol, salicylate, and acetaminophen level were unremarkable. The urine drug screen was positive for tetrahydrocannabinol (THC) (cutoff value: 50 ng/mL). Electrocardiogram (EKG) revealed sinus bradycardia with first-degree AV block with a PR interval of 184 ms (Figure 1). The polymerase chain reaction test for coronavirus disease 2019 was also negative. The patient was referred to poison control, and observation and monitoring were suggested. The cardiologist was also consulted and advised to repeat the EKG before discharge.

The patient was admitted and placed on continuous cardiac monitoring. His vitals were stable and his heart rate was normal upon admission (four hours after ingestion). After nine hours of admission (12 hours after ingestion), the patient was back to his usual state and was active and playful. EKG was done before discharge and revealed normal sinus rhythm with a PR interval of 124. A social worker was involved in the care of the patient and counseling regarding child safety was done. The child was discharged after 36 hours of hospital stay.

## Discussion

According to the United Nations World Drug Report 2021, 200 million people or 4% of the global population use cannabis (marijuana), making it the most abused illicit substance. The same applies to the United States where over 29 million people aged 18 and older were estimated to be using cannabis in the past month [1]. With the two-fold rise in cannabis use in the past decade and the decriminalization of marijuana in the United States, cannabis exposure in children has increased [2].

From 2017 to 2019, data obtained from poison centers in the United States revealed that there were 4,172 calls involving cannabis exposures for children aged zero to nine years. Children aged three to five years had the highest proportion of all exposures (43%), and most cases were exposed by ingestion (72%). In addition, calls due to edible cannabis ingestion were greater in states where marijuana is legal [3]. Exposure at a young age, the increasing potency of cannabis products in the market, and the availability of edibles that are appealing to children raise an issue of public health concern [13].

Various preparations of cannabis edibles (brownies, cookies, candy, gummies, etc.) are currently available in the US market. After ingestion, absorption is slow and variable due to the degradation by gastric acid and hepatic first-pass metabolism. Bioavailability ranges from 10% to 20% and is lower than the inhaled route [14,15]. However, many edibles contain a much higher concentration of THC than smoked cannabis [15]. In addition, edibles are ingested by a greater number of children due to their palatability, interesting packaging, and delayed onset of symptoms [3,16,17]. Peak concentration, which reflects the onset of clinical manifestations, is achieved in one to two hours but may be delayed up to eight hours [14,16]. In this case, the patient became symptomatic one hour after the consumption of gummies, and the symptoms lasted approximately 12 hours. This is compatible with the existing literature in which the duration of clinical effects of cannabis ingestion in children lasted from two to 24 hours [9].

Numerous well-documented studies of cannabis ingestion in children are available in the literature. Similar to the case presented, most ingestions occur in children less than five years old [4-8]. It typically occurs unintentionally after exploratory ingestion of marijuana intended for adult consumption [18]. The most common presenting symptoms are drowsiness, lethargy, or somnolence, which were also observed in the index patient. Other commonly reported signs and symptoms include ataxia, tachycardia, and hypotonia. Severe symptoms requiring intensive care such as respiratory depression, coma, and seizures have also been reported [4-8].

Clinical manifestations of marijuana toxicity can be attributed to its action on the cannabinoid 1 (CB1) receptors predominantly expressed in the central nervous system [14]. CB1 receptors are also present in the cardiovascular system, including the myocardium, vascular endothelial cells, and smooth muscle cells, and in the peripheral nervous system, including vagal afferent neurons [18]. In the pediatric population, tachycardia is the most common cardiovascular symptom [4,5]. Tachycardia results from sympathetic stimulation and can also be due to reflex tachycardia from vasodilation secondary to parasympathetic stimulation [11,12]. Bradycardia, which was observed in this case, is an uncommon cardiovascular manifestation in children with marijuana exposure. Incidence is not well established but from the studies reviewed, it can be estimated to range from 0.4% to 4% [19]. Both bradycardia and hypotension can result from increased cardiac vagal tone, especially with higher doses of exposure [11]. This could mean that the child in this case must have ingested a high dose of THC to elicit a parasympathetic response.

In this case, an EKG done to further investigate bradycardia revealed a prolonged PR interval (180 ms, upper limit for age is 150 ms) [9], consistent with a first-degree AV block. The incidence of AV block and other arrhythmias in children with cannabis exposure is unknown. In one multicenter retrospective study among 876,431 teenagers with cannabis use disorder, 4,043 (0.5%) had arrhythmia. The most frequent cardiac arrhythmia observed was atrial fibrillation (0.1%), followed by Wolff-Parkinson-White syndrome and ventricular tachycardia (0.08%) [20]. In another retrospective study, two adolescents had first-degree AV block and one had second-degree AV block among 174 children with a positive urine drug screen for cannabis [21].

The exact mechanism of cardiac bradyarrhythmia in cannabis exposure has not been fully elucidated. One theory that can explain the slowing of conduction across the AV node is the activation of CB1 receptors leading to sympathetic inhibition and enhanced vagal tone [22]. With chronic cannabis use, modulation of the autonomic nervous system causes diminished sympathetic activation and increased parasympathetic response [22]. However, no studies have yet proven that chronic second or third-hand cannabis smoke exposure can lead to such autonomic nervous system changes. Resolution of the AV block within 24 hours in the case presented may signify that this finding could be transient and supportive measures are sufficient for its management.

Cannabis-induced AV block remains to be a diagnosis of exclusion. After a detailed history, other notable causes of acquired cardiac conduction abnormalities that were excluded were infection, hypoglycemia, electrolyte imbalance, and other substances which might be co-ingested by the child (salicylate, acetaminophen, ethanol) [22]. The sudden onset of drowsiness, bradycardia, and first-degree AV block in a previously healthy toddler positive for THC in the qualitative urine drug test, as well as the resolution of symptoms after exposure was discontinued, were all suggestive of the causal role of cannabis in the child’s symptomatology.

## Conclusions

The increasing incidence of cannabis ingestion in young children is an issue of public health concern. Symptoms can be mild to life-threatening and can involve multiple organ systems. Cardiovascular manifestations such as heart rate, rhythm, and blood pressure abnormalities can be observed. Although bradycardia and bradyarrhythmia are uncommon after consumption of cannabis in children, clinicians should be aware of these findings and must consider evaluating for marijuana toxicity whenever presented with these acute signs in a setting of possible exposure. Treatment is mainly supportive and should be focused on ensuring the child’s safety even after discharge.
